# Transamniotic mesenchymal stem cell therapy for neural tube defects preserves neural function through lesion-specific engraftment and regeneration

**DOI:** 10.1038/s41419-020-2734-3

**Published:** 2020-07-13

**Authors:** Xiaowei Wei, Wei Ma, Hui Gu, Dan Liu, Wenting Luo, Yuzuo Bai, Weilin Wang, Vincent Chi Hang Lui, Peixin Yang, Zhengwei Yuan

**Affiliations:** 1 Key Laboratory of Health Ministry for Congenital Malformation, Shengjing Hospital, China Medical University, Shenyang, China; 2 Department of Pediatric Surgery, Shengjing Hospital, China Medical University, Shenyang, PR China; 3 Department of Surgery, Li Ka Shing Faculty of Medicine, The University of Hong Kong, Pok Fu Lam, Hong Kong China; 4 Departments of Obstetrics, Gynecology, and Reproductive Sciences, University of Maryland School of Medicine, Baltimore, MD 21201 USA

**Keywords:** Diseases of the nervous system, Neural tube defects

## Abstract

Neural tube defects (NTDs) lead to prenatal mortality and lifelong morbidity. Currently, surgical closure of NTD lesions results in limited functional recovery. We previously suggested that nerve regeneration was critical for NTD therapy. Here, we report that transamniotic bone marrow-derived mesenchymal stem cell (BMSC) therapy for NTDs during early development may achieve beneficial functional recovery. In our ex vivo rat embryonic NTD model, BMSCs injected into the amniotic cavity spontaneously migrated into the defective neural tissue. Hepatocyte growth factor and its receptor c-MET were found to play critical roles in this NTD lesion-specific migration. Using the in vivo rat fetal NTD model, we further discovered that the engrafted BMSCs specifically differentiated into the cell types of the defective tissue, including skin and different types of neurons in situ. BMSC treatment triggered skin repair in fetuses, leading to a 29.9 ± 5.6% reduction in the skin lesion area. The electrophysiological functional recovery assay revealed a decreased latency and increased motor-evoked potential amplitude in the BMSC-treated fetuses. Based on these positive outcomes, ease of operation, and reduced trauma to the mother and fetus, we propose that transamniotic BMSC administration could be a new effective therapy for NTDs.

## Introduction

Neural tube defects (NTDs) resulting from incomplete closure of the neural tube are the second most common congenital anomalies that currently have limited therapeutic benefits^1^. Globally, 300,000–400,000 babies are born with NTDs yearly^2^. Prenatal open surgical, foetoscopic, or mini-hysterotomy interventions have been performed to cover the neural tube lesion and prevent further neurodegeneration^3–7^. These clinical trials showed that compared to postnatal surgery, fetal surgery at 19–26 weeks of gestation reduces hindbrain herniation, decreases the need for ventriculoperitoneal shunt, and improves lower extremity function. The long-term neurological outcomes after fetal surgery have also been confirmed^8–10^. However, even after fetal surgery, the majority of individuals with NTDs continue to experience sensory and motor weakness in the legs, fecal or urinary incontinence, and hindbrain herniation after birth^7,8^. The total cost of care for each person with NTDs is estimated to be $791,900^11^. In addition, prenatal surgery confers increased maternal-fetal risk of preterm birth, intraoperative complications, peripartum bleeding, and uterine dehiscence at delivery^7,8^. We have previously shown that deficiencies in the sensory, motor, and parasympathetic neurons are the primary anomalies coexisting with spinal malformations in fetal rats with NTDs^12–14^. Thus, prenatal therapy for NTDs will probably progress from solely covering the NTD to a new strategy for the regeneration and further protection of the affected neurons.

Bone marrow-derived mesenchymal stem cell (BMSC)-based therapies are widely considered to treat neurological disorders and have undergone improvements for the treatment of various neurodegenerative diseases, including Parkinson′s disease^15^, multiple sclerosis^16,17^, stroke^18^, and acute brain or spinal cord insults^19^. The clinical utility of BMSCs is owed to their convenient isolation, capacity of self-renewal, low immunogenicity, multi-lineage differentiation, and safety and feasibility for transplantation into animal models or humans. As deficiencies resulting from NTDs are found in the neural tissue, skin, muscle, and skeleton, BMSCs with multi-lineage differentiation potential could facilitate the repair of multiple tissue damages resulting from NTDs. Indeed, our previous studies have shown that BMSCs directly injected into the fetal spinal column differentiated into nerve and muscle cells, secreted growth factors, and reduced neural apoptosis^20–24^. Nevertheless, direct cell injection into neural tissue is not only a technical challenge but also poses a risk of trauma to the fetus. Moreover, prenatal cell transplantation surgery is typically performed during late pregnancy, when the pathology is already evident and the neural damage caused by NTDs may be irreversible^25^.

In utero delivery of reagents (e.g., drugs, genes, or antisense oligonucleotides) to the amniotic cavity has been considered an effective therapeutic approach for the treatment of disorders in early embryos/fetuses^26–28^. Transamniotic injection allows delivering stem cells before the formation of irreversible damage, and the rapid growth of early embryos is ideal for the engraftment and differentiation of stem cells. As the embryo/fetus is surrounded by amniotic fluid, the defective neural tube is directly exposed to the injected BMSCs and, therefore, is a prime target for repair. Research has shown that transamniotic injection of stem cells, including neural stem cells (NSCs) and amniotic fluid-derived and placenta-derived MSCs (afMSC and pMSCs), is a promising treatment in animal models of congenital diseases^29–37^. These studies demonstrated that after transamniotic injection, afMSC and pMSCs robustly migrated into the fetal bone marrow, placenta, umbilical cord, and spinal cord lesion^30,31,35–37^. Moreover, transplanted MSCs induced varying degrees of coverage of the defects through a membrane that appeared to be rudimentary skin based on histological analysis^32,37^. In turn, the coverage attenuated the Chiari-II malformation in rat fetuses with spina bifida^33^. However, these studies did not dynamically track the migration of donor MSCs in living embryonic rats, and the neurological function recovery remained unaddressed.

Here, we used ex vivo and in vivo rat embryonic/fetal NTD models to examine whether transamniotic BMSC injection could specifically and effectively repair NTDs during early-stage development. This study may provide important additional experimental basis for the clinical use of transamniotic stem cell transplantation.

## Results

### All-trans retinoic acid (atRA)-induced ex vivo NTD model

**Fig. 1:**
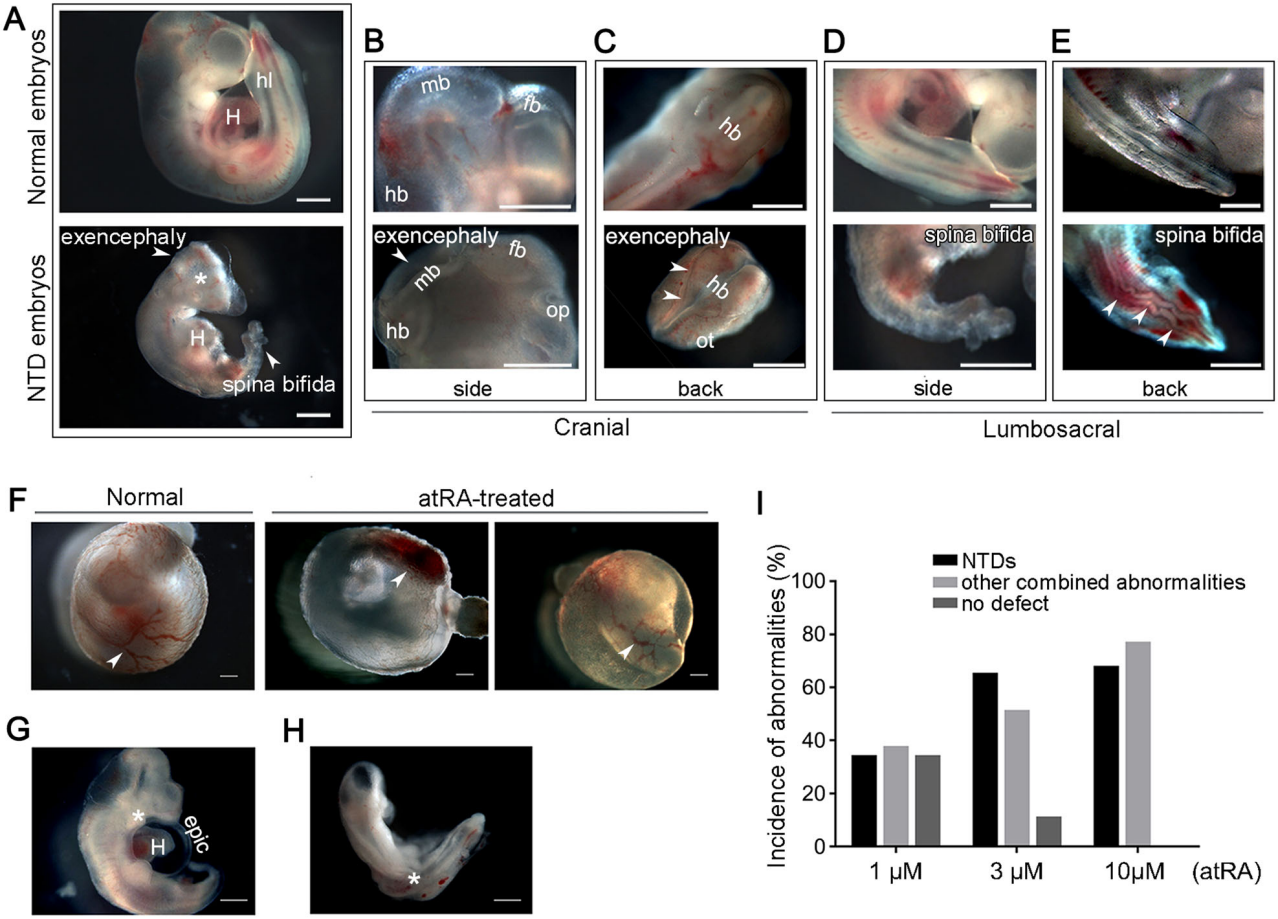
atRA induces NTDs in rat embryos ex vivo. Abnormalities were observed in the craniocerebral and sacrococcygeal regions of atRA-treated embryonic rats. **a** Representative photos of normal and NTD embryos under a stereological microscope. Compared to the normal embryo, the NTD embryo exhibited distinct exencephaly (arrowhead), spina bifida (arrowhead), and craniofacial deformity (asterisk). **b** Magnified side view of the brains of normal (upper) and exencephalic (lower, arrowhead) embryos. **c** Magnified view from the back of the brain of normal (upper) and exencephalic (lower, arrowheads) embryos. **d** Magnified side view of the lumbosacral regions: the closed neural tube from a normal embryo (upper) and a defective neural tube from an embryo with spina bifida (lower). **e** Magnified view from the back of the lumbosacral region. The spinal neural tube of the normal embryo was smooth and continuous (upper) whereas the spinal neural tube of the spina bifida embryo was disturbed and not completely closed (lower, arrowheads). **f** The yolk sacs of normal and atRA-treated malformed embryos. A well-formed extensive blood vessel network with blood circulation (arrowhead) was observed in the yolk sac of the normal embryo. An under-developed blood vessel network (arrowhead) was observed in the yolk sac of the atRA-treated embryo. **g** A representative atRA-treated embryo with delayed formation of the heart chamber, detachment of the epicardium, and branchial arch malformations (asterisk). **h** A representative atRA-treated embryo with body-axis dysplasia (asterisk). **i** The relationship between the atRA dose and embryo dysmorphology. Epic epicardium, fb forebrain, hb hindbrain, hl hind lim, H heart, mb midbrain, ot otic vesicle, op optic vesicle. Scale bars: 500 μm.

We established the rat embryo ex vivo NTD model by adding various concentrations of atRA to the whole embryo culture (WEC) system. Three micromolar atRA was the optimal teratogenic dose (Fig. 1), inducing mainly spina bifida and exencephaly (Fig. 1a–e). In addition, hypoplastic branchial arch, atelocardia, incomplete turning of neural axis, and other defects were observed in atRA-treated embryos (Fig. 1f–i).

### NTD lesion-specific migration of transamniotically injected BMSCs

**Fig. 2:**
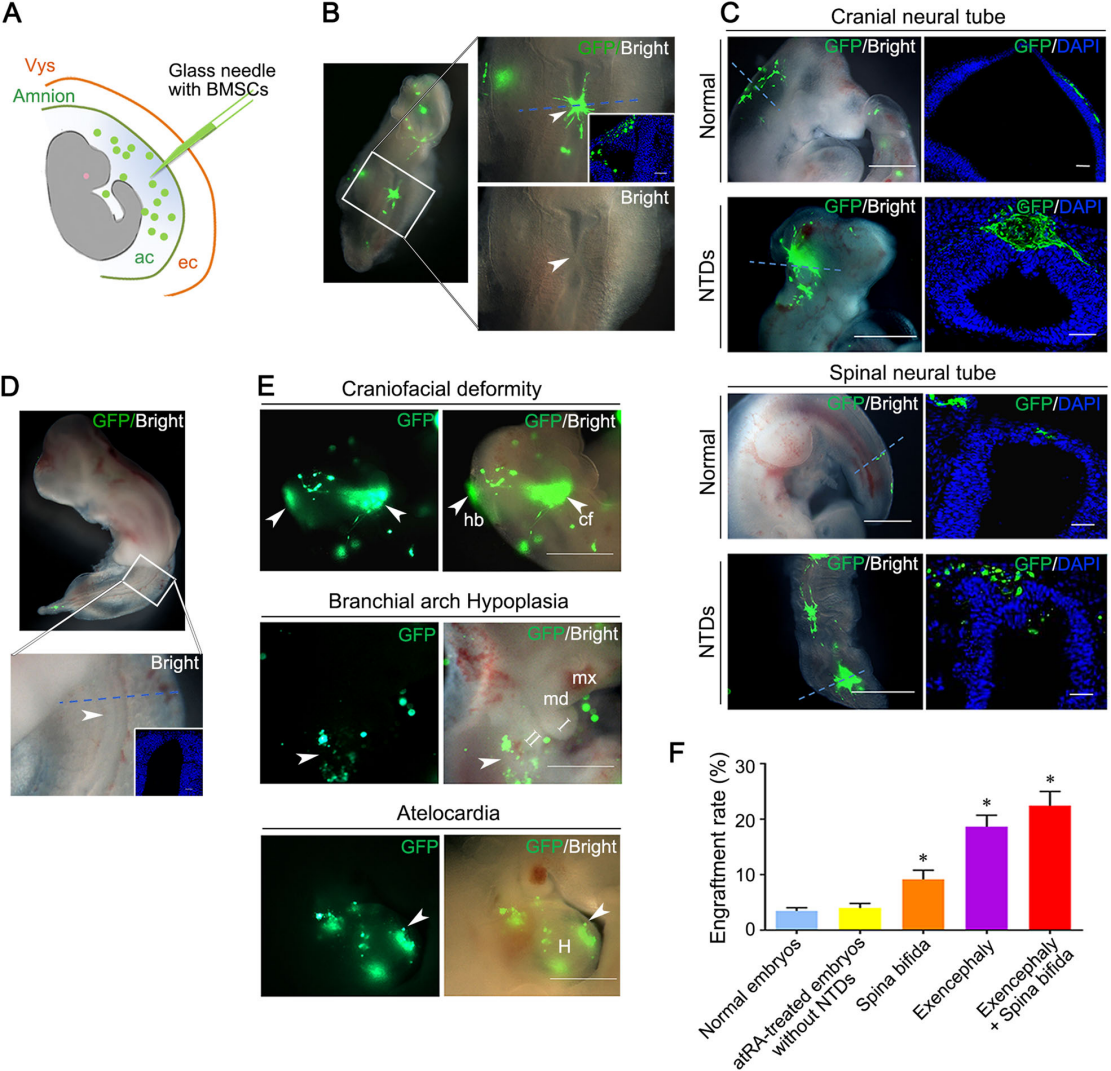
GFP-labeled BMSCs specifically engraft into NTD-lesions. **a** A schematic picture of transamniotic BMSC microinjection of an E10 embryo. The microinjection pipette (blue) traversed the visceral yolk sac (vys, orange), exocoelomic cavity (ec) and finally the amnion (green) before entering the amniotic cavity (ac). **b** GFP^+^ BMSCs (green) distributed specifically at defective regions of the neural tube after transamniotic BMSCs transplantation. The arrowhead indicates a closed neural tube region. Inset: transverse section along the blue dotted line. Nuclei were stained blue with DAPI. **c** Representative fluorescent stereomicroscopic and microscopic images of embryos with BMSCs engraftment and their transverse sections. Scale bars: 500 μm (fluorescent stereomicroscopic images) and 50 μm (cryosection images). **d** Normal embryo with a closed neural tube after BMSCs transplantation. **e** GFP^+^ BMSCs detected in the abnormal regions of embryos with other deformities. Arrowheads indicate typical deformities where BMSCs were engrafted. Scale bars: 500 μm. cf craniofacial, hb hindbrain, H heart, md mandible, mx maxilla. **f** Evaluation of the BMSC engraftment rate in different types of NTDs (Controls, *n* = 12; atRA-treated embryos without NTDs, *n* = 11; Spina bifida, *n* = 11; Exencephaly, *n* = 9; Exencephaly+ spina bifida, *n* = 9). Engraftment rates were determined by the total number of GFP^+^ BMSCs in continuous frozen sections/ the number of transplanted BMSCs at 48 h after transplantation. * indicate significant difference compare with those in normally developed embryos, *P* < *0.05*.

We harvested 210 normal and 232 atRA-treated malformed embryos following transamniotic injection (Fig. 2a), finding that the transplanted BMSCs spontaneously migrated into defective regions of the neural tube in 87.1% (202/232) of the malformed embryos. The open neural tubes were closed at the site of BMSC engraftment in 26.7% (54/202) of these embryos (Fig. 2b). Most transplanted BMSCs were localized at the dorsal midline to the roof plate of the defective neural tube (Figs. 2c and S1A), peripheral mesenchyme (Fig. S1B), and ganglion (Fig. S1C). Only a few transplanted BMSCs (Green Fluorescent Protein positive, GFP^+^) were found dispersed in the neural tubes of normal embryos (Fig. 2d). GFP^+^ BMSCs were also detected at the abnormal craniofacial region, branchial arch agenesis, and hypoplastic heart (Fig. 2e). The engraftment of GFP^+^ BMSCs was significantly higher in NTD embryos than in normal embryos (*P* < 0.01) (Fig. 2f). No significant difference was observed between BMSC engraftment rates of normal atRA-treated and control embryos (*P* = 0.441), indicating that atRA did not influence BMSC engraftment. To observe the potential effects of BMSCs on embryonic development, sterile phosphate buffered saline (PBS) or BMSCs were transamniotically delivered to E10 embryos (*n* = 12/group). No significant differences in crown-rump length, head length, yolk sac diameter, somite number, or total morphological score were observed between the not injected, PBS-injected, and BMSC-injected groups (Table S1).

**Fig. 3:**
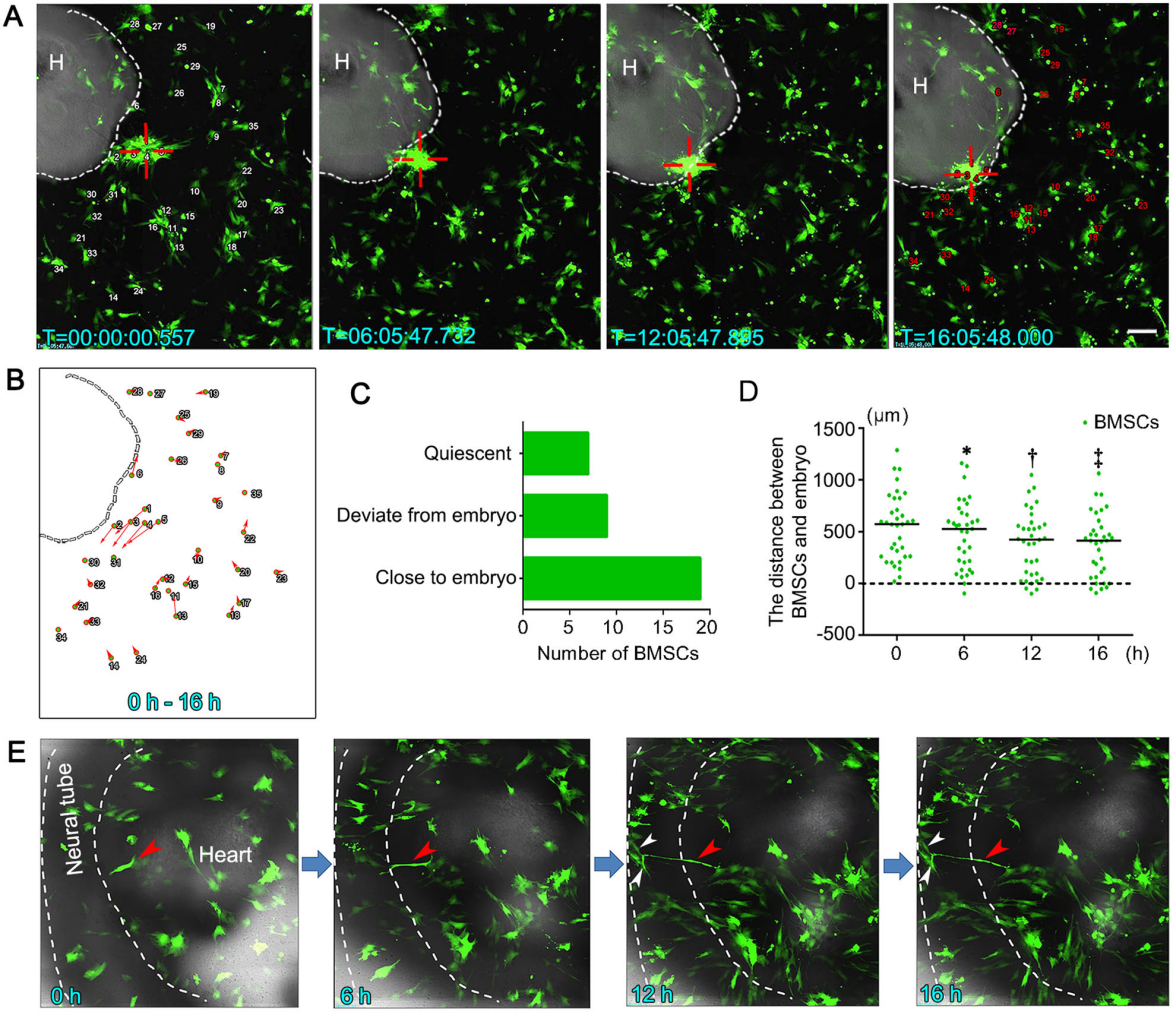
Time-lapse tracking of BMSCs co-cultured with atRA-treated NTD embryos. **a** Representative time-lapse live cell images of BMSC migration. The white dotted line demarcates the edge of the embryo. Red hollow crosses indicate that the BMSCs migrated spontaneously into the neural tube. The numbers indicate the 35 cells used for cell tracing analysis. **b** The tracing analysis cell movement for 35 cells monitored from 0 to 16 h. The small circles represent the starting positions of the cells, and the arrows indicate the direction and distance traveled by the cells. **c** Histogram of cell numbers of three migration patterns. **d** The distance between the BMSCs and the embryo versus culture time. The median distance at each time point is indicated by the short black line. * indicate significant difference compared with 0 h, † indicate significant difference compared with 6 h, ‡ indicate significant difference compared with 12 h, *P* < *0.05*. **e** The migrating BMSCs (red arrowhead) transformed into mature neuron-like cells with long axons that made contact with other BMSCs (white arrowheads). Scale bars: 200 μm.

To further confirm the NTD lesion-specific engraftment of GFP^+^ BMSCs, we executed time-lapse tracking. Results demonstrated that some BMSCs co-cultured with atRA-treated NTD embryos gathered and migrated to the defective neural tube (Fig. 3a and Video S1). To quantify the directional movement of BMSCs, we tracked the migration of 35 randomly selected BMSCs surrounding the NTD embryo and observed that 19 (54.3%) of them moved towards the embryo (Fig. 3b, c). The median distances between the tracked BMSCs and the embryo were shortened after co-culturing for 16 h (Fig. 3d). In addition, a few BMSCs that had engrafted in the neural tube transformed into neuron-like cells and extended long, dendrite-like cellular processes connecting with neighboring cells (Fig. 3e and Video S2).

### The primary role of chemotactic hepatocyte growth factor (HGF)/c-MET pathway in lesion-specific migration of BMSC

**Fig. 4:**
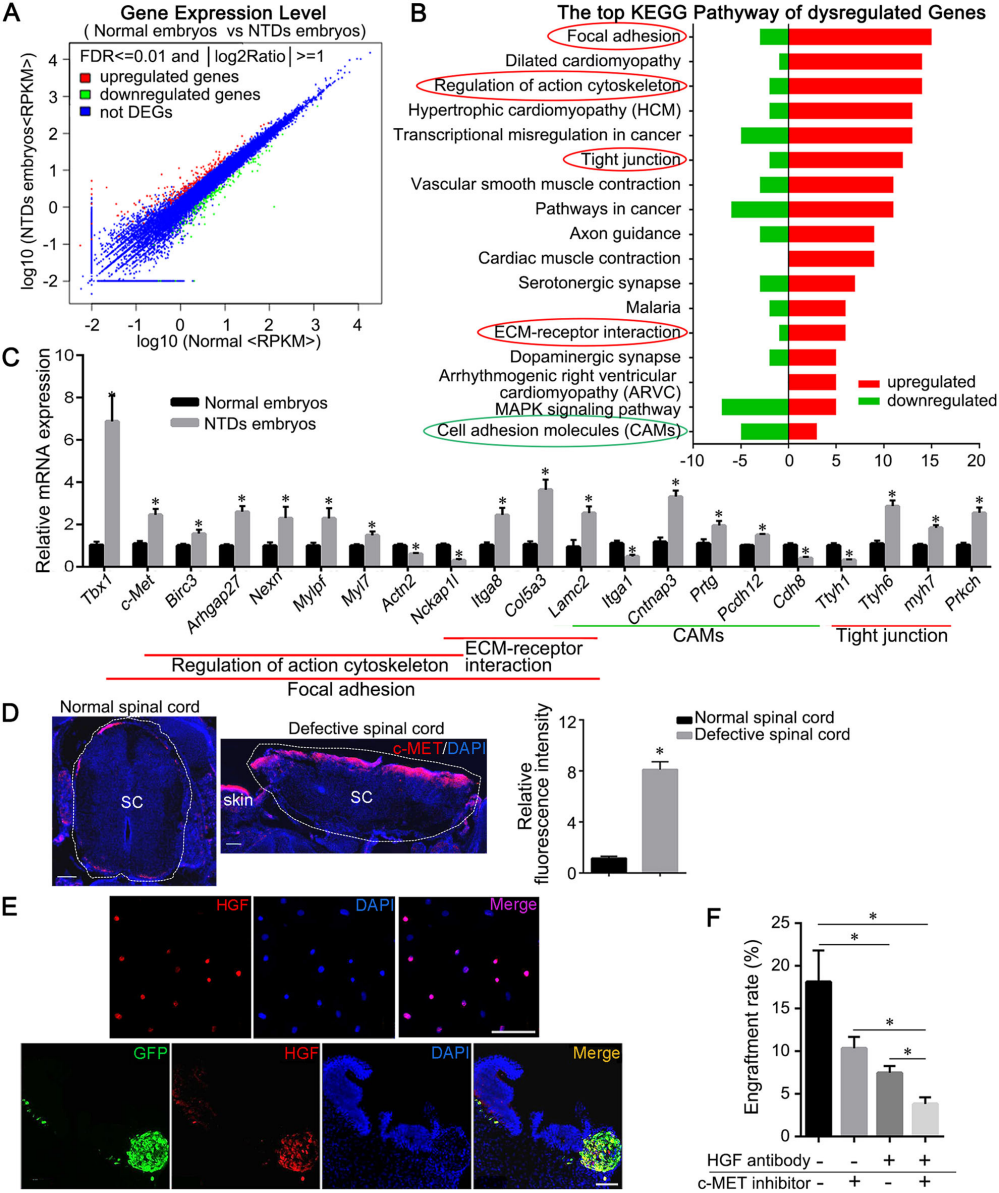
Differentially expressed genes and KEGG pathways in NTD embryos. **a** Scatter plot of differentially expressed mRNAs between the NTDs and normal rat embryos. Red dots indicate upregulated mRNAs, blue dots indicate equally expressed mRNAs, and green dots indicate downregulated mRNAs. The cut-off criteria were FDR ≤ 0.001 and |log2Ratio|≥ 1. Ratio = NTD embryos-RPKM/normal embryos-RPKM. **b** KEGG pathway analysis of differentially expressed genes (DEGs). The signaling pathways included the focal adhesion pathway, regulation of actin cytoskeleton pathway, tight junction pathway, ECM-receptor interaction pathway, and cell adhesion molecular pathway. The pathways of interest are indicated with circles. The red circles indicate the pathways with more upregulated genes. The green circle indicates the pathway with more downregulated genes. **c** Relative mRNA expression of the differentially expressed genes with |log2 fold change|>0.5, fold change = NTD embryos /normal embryos. (*P < 0.05, NTD embryos versus normal embryos). The expression levels were normalized to *Gapdh*. **d** Anti-c-MET (red) labeling of normal and defective spinal cords. The histogram shows the relative fluorescence intensities of c-MET in defective spinal cords compared with those of normal spinal cords (*P < 0.05, 10× field, *n* = 6). **e** Anti-HGF labeling in BMSCs cultured in vitro (top row) and engrafted GFP^+^ BMSCs in defective spinal cords following transplantation (bottom row). **f** Engraftment rate analysis for BMSCs, incubated without (–) or with (+) anti-HGF antibody for 1 h prior to BMSC transplantation into the amniotic cavity of NTD embryos without (–) or with (+) c-MET inhibitor treatment (*P < 0.05, *n* = 6 in per group). Scale bars: 100 μm.

As BMSCs specifically migrated to NTD lesions after transamniotic injection, we speculated that migration-related factors expressed in the defective neural tube might help recruiting BMSCs. To investigate this hypothesis, Ultra-high Throughput Sequencing Analysis of mRNA-derived cDNA Libraries (RNA-seq) was used to identify differentially expressed genes between normal and defective neural tubes, focusing on key factors involved in cell homing and migration. The differential expression analysis identified 192 upregulated and 155 downregulated genes in spinal neural tubes of NTD embryos compared to normal controls (Fig. 4a). Pathway enrichment analysis demonstrated that 28 pathways were dysregulated in NTD embryos (Table S2). These pathways were implicated in cell chemotactic migration with the focal adhesion, actin cytoskeleton, tight junction, and extracellular matrixc (ECM)-receptor pathways upregulated and the cell adhesion molecule pathway downregulated in NTD embryos (Fig. 4b).

We further identified 32 differentially expressed genes implicated in cell chemotactic migration by real-time Quantitative Reverse-transcription PCR (RT-qPCR), which confirmed the RNA-seq results for 27 of these genes. Twenty-one genes had a|log2 fold change|> 0.5 (Fig. 4c). A significant positive correlation between the RNA-seq and RT-qPCR datasets based on Spearman correlation analysis (*r* = 0.80, *P* < 0.01) was observed. Next, we assessed the levels of proteins encoded by three genes (*c-Met, Tbx1, and Itga1*) involved in cell migration^17,38,39^ that presented highly significant differences in their expression. We found that c-MET was significantly overexpressed in the damaged spinal neural tube (Fig. 4d), and changes in TBX1 and ITGA1 protein levels were not significant. c-MET is the primary receptor of HGF, and HGF/c-MET signaling is involved in cell chemotactic migration and neuroprotection^17,37^. Thus, we determined HGF protein levels in both cultured BMSCs and developmental neural tubes. HGF was highly expressed in 66.3 ± 3.2% of the cultured BMSCs (Fig. 4e). High HGF-expressing GFP^+^ BMSCs engrafted into high c-MET-expressing areas of the defective neural tube (Fig. 4d, e). To further verify whether the HGF/c-MET pathway plays a critical role in NTD lesion-specific migration of BMSCs, an anti-HGF antibody or c-MET inhibitor was used to inhibit the activity of HGF in BMSCs or c-MET in the defective neural tube before transplantation, respectively. The BMSC engraftment rate was significantly lower in the double inhibition group than in the single inhibition or control groups (*P* < 0.05) (Fig. 4f).

### Multi-lineage differentiation of transplanted BMSCs in defective regions of NTDs

**Fig. 5:**
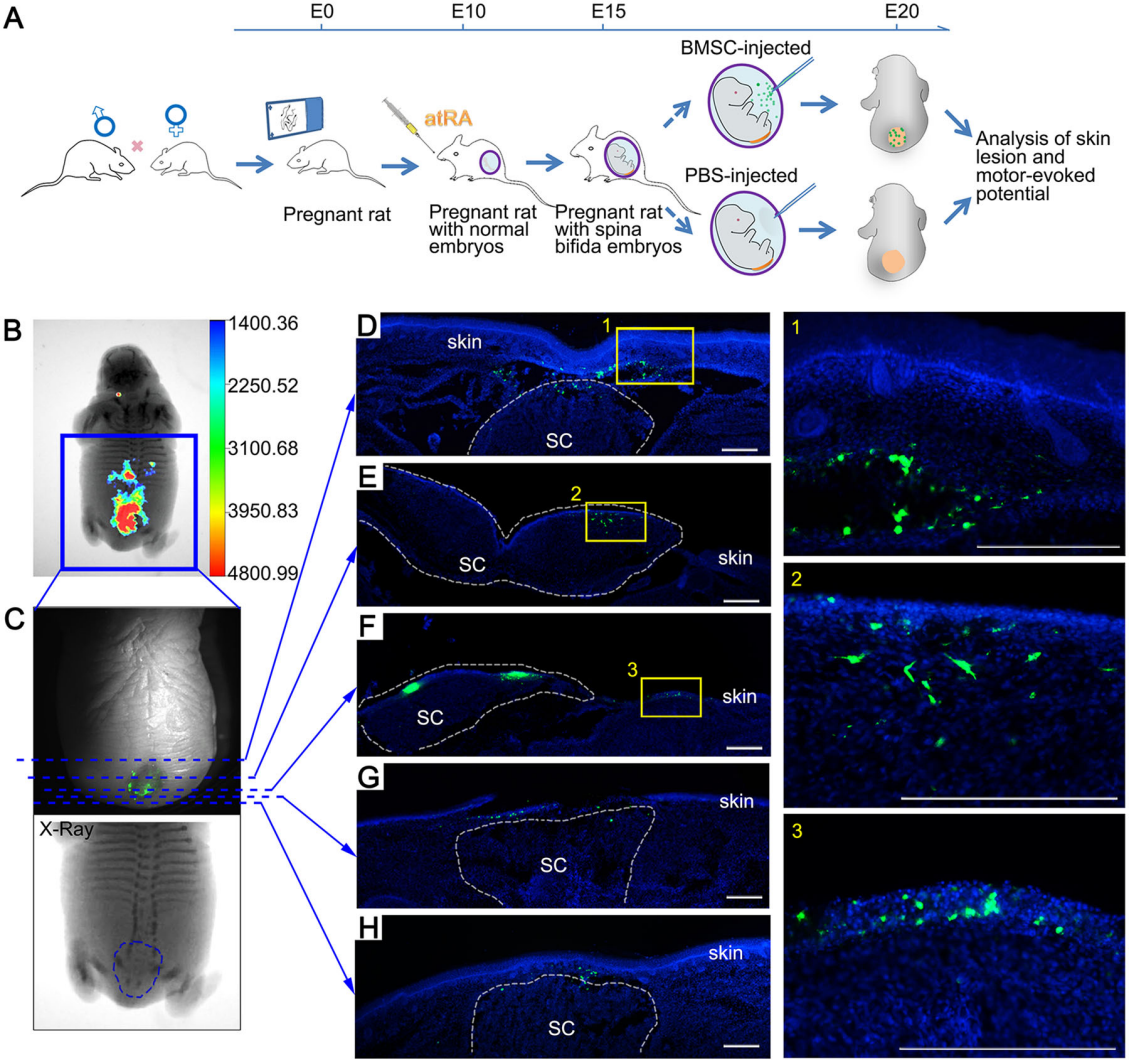
Transplanted BMSCs engrafted in different tissues of the lesion. **a** Experimental design and time course: intragastric atRA administration was performed on the morning of E10, the intrauterine BMSC microinjection was performed on E15, and analysis of skin lesions and motor-evoked potential (MEPs) of the fetuses were performed after cesarean section on E20. **b** Representative overlay images of X-ray and fluorescence images show a large number of GFP^+^ BMSCs at the defective lumbosacral region. **c** The fluorescence (top) and X-ray (bottom) images were enlarged from the blue box in A. The blue dotted lines indicate where the fluorescence sections (**d**–**h**) were obtained and the blue dotted circle indicates the abnormal lumbar sacrum in the fetus. **d**–**h** Representative sections of BMSC engraftment from upper edge of the lesion to the lower edge. The GFP^+^ BMSCs (green) scattered on the subcutaneous tissue of the defect (**d** and **h**), dorsal view of the defective spinal cord (**e** and **f**), abnormal skin surface (**f**), and the place where the skin tissue should be extended to cover the defect (**g**). Highlighted regions in **d**–**f** were magnified and shown on the right. Scale bars: 100 μm.

To determine the repair effect of BMSC transplantation in vivo, BMSCs were injected into the amnion sac of E15 embryos of anaesthetized pregnant rats (Fig. 5a). The distribution of the transplanted BMSCs in E20 fetuses was observed using an in vivo imaging system, which detected GFP^+^ BMSCs in deep tissues covered by the repaired skin. Consistent with our ex vivo findings, transamniotically transplanted BMSCs migrated to the dorsal region of defective spinal cords (Fig. 5b, c), and numerous GFP^+^ BMSCs were detected in the defective spinal cord under the repaired skin. This analysis also confirmed serious, not mild, defects in the spinal cord before BMSC transplantation (Fig. 5c). Scanning of all serial sections of whole fetuses revealed that GFP^+^ BMSCs mainly engrafted on the surface of the damaged tissue, with some cells being integrated into the repaired skin (Fig. 5f), spinal cord (Fig. 5e and g), and subcutaneous tissue (Fig. 5d and h). Few GFP^+^ cells were detected at other sites (Fig. S2). These images suggest that the transplanted BMSCs specifically migrate and integrate into the deformed tissues, where they might be involved in skin and neural repair processes. Moreover, we scanned PBS-injected fetuses from the same dams to investigate possible contaminations with transplanted (GFP^+^) BMSCs through the circulatory system or other means, but no GFP signal was detected in those.

**Fig. 6:**
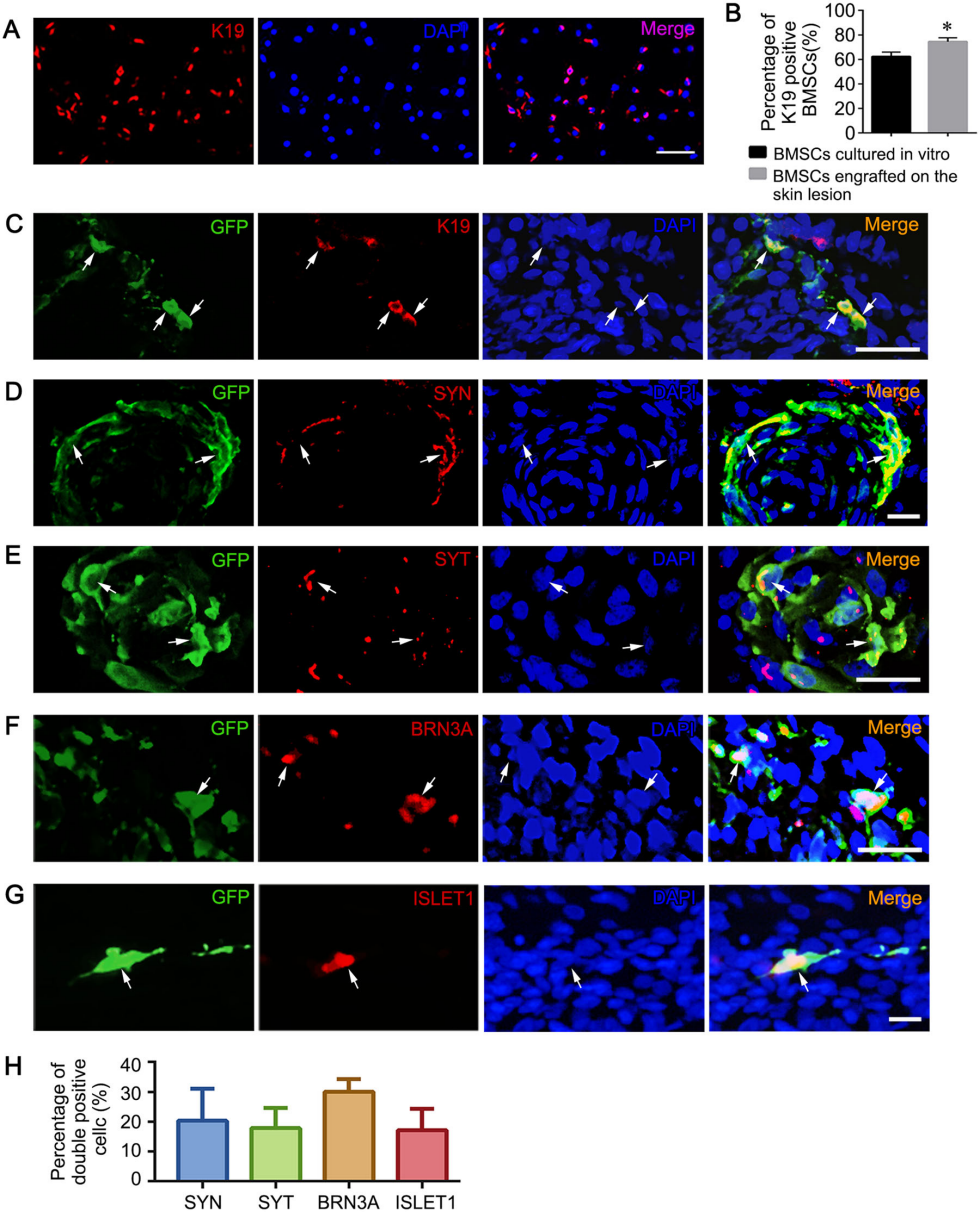
In utero transamniotic transplanted BMSCs differentiate into multi-lineage cells. **a** Immunofluorescence staining for K19 (red) in cultured BMSCs. **b** Percentage of K19^+^ BMSCs in vitro and in the abnormal skin region of fetuses. *Significant difference compare to BMSCs cultured in vitro group, *P* < 0.05. **c** Dual-labeling for K19 (red) and GFP (green) in the tissue sections of the defective spinal cord after BMSC transplantation. White arrows denote the localizations of representative double-positive cells. **d**–**g** Representative immunofluorescence images showing transplanted BMSCs expressing SYN (**d**), SYT (**e**), BRN3A (**f**), and ISLET1 (**g**) in the defective spinal cord. Scale bars: 50 μm. **h** Quantitative analysis of SYN, SYT, BRN3A, and ISLET1 in transplanted BMSCs in defective spinal cords. The percentage of BMSCs expressing these markers were determined as the number of double positive cells/total GFP positive cells (40× field, *n* = 10).

The differentiation potential is a key mechanism of MSC-mediated therapy^40^. Previously, we demonstrated that BMSCs directly injected into the spinal column are mainly located in the deep layer of the defective neural tissue and express specific protein markers of many neural cells^20–22^. Here, we observed a more widespread distribution of transamniotically transplanted BMSCs in NTD fetuses and regeneration of the defective skin. Compared to BMSCs in culture, the expression of K19, a specific marker of epidermal stem cells, was significantly increased in BMSCs engrafted in the defective skin (*P* < 0.05) (Fig. 6a–c).

In utero transamniotic transplantation of BMSCs prolonged the cell differentiation duration. Here, we assessed the expression of several mature neuronal markers in BMSCs engrafted into the spinal cord. Results showed that 20.3 ± 3.7% and 17.8 ± 3.2% of the engrafted BMSCs expressed SYNAPSIN1 (SYN) and 17.8 ± 3.2% expressed SYNAPTOTAGMIN (SYT), respectively, indicating that some transplanted BMSCs may establish synaptic contacts with surrounding endogenous spinal cord cells (Fig. 6d, e, and h). GFP^+^ BMSCs were also detected around the dorsal root ganglion, an area enriched with sensory neurons. Therefore, we examined the expression of sensory neuron markers in defective spinal cords following BMSC transplantation and found that 30.1 ± 1.8% of the transplanted BMSCs expressed BRN3A (Fig. 6f and h). ISLET1 is required for motor neuron development and is expressed in all post-mitotic motor neurons, which are usually distributed in the ventral side of the developing spinal cord^41^. We found that ISLET1 was expressed in 17.2 ± 3.1% of GFP^+^ BMSCs engrafted into spinal cords (Fig. 6g, h).

### Skin repair and neural function recovery in NTD fetuses by injected BMSCs

**Fig. 7:**
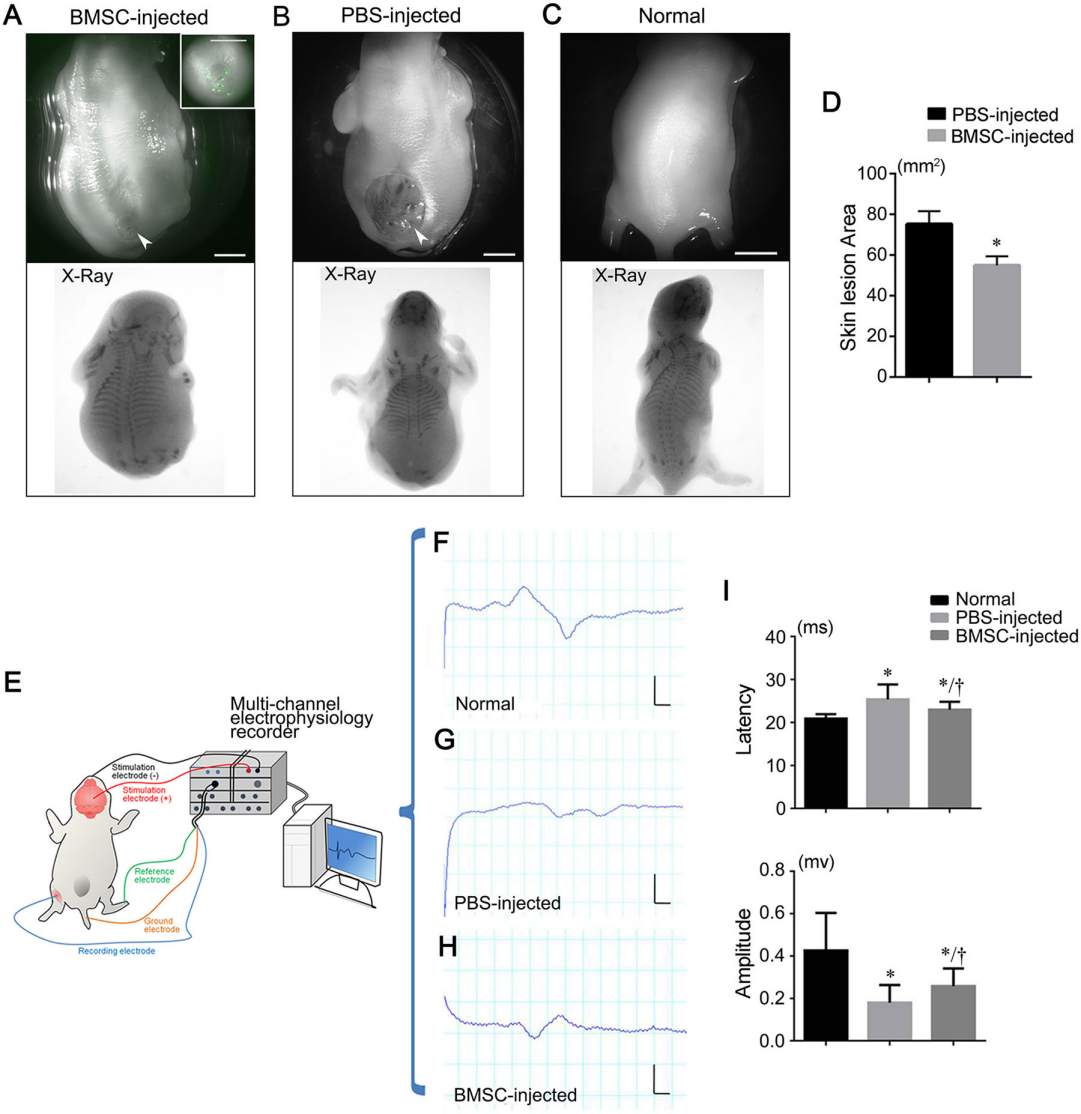
Skin repair and neural function recovery in NTD fetuses after BMSC transplantation. **a** Representative images of E20 rat fetuses with spina bifida aperta after BMSC transplantation taken using fluorescent stereomicroscopy (top) and in vivo imaging (bottom). The lumbosacral region (arrowhead) is enlarged in the inset. **b** Untreated spina bifida aperta fetuses on E20 were examined by stereomicroscopy and in vivo imaging. The arrowhead indicates the unclosed lumbosacral spinal cord and skin. The presence of skeletal defects at the lumbosacral regions were shown by X-ray imaging. **c** Stereomicroscopic (top) and X-ray images of a normal rat fetus. **d** Quantitative analysis of the skin lesion area comparing the BMSC-injected (*n* = 30) and PBS-injected groups (*n* = 23). * *P* < 0.05 compare to PBS-injected group, Student′s *t*-test. **e** The operation schematic diagram of MEPs experiments on rat fetuses. **f**–**h** Representative MEP waveforms collected by a multi-channel electrophysiology recorder from a normal fetus (**f**), a fetus with spina bifida aperta (**g**), and a fetus with spina bifida aperta that received transplanted BMSCs (**h**). **i** Analysis of MEP latency and amplitude in the normal controls (*n* = 10), PBS*-*injected group (*n* = 12) and BMSC-injected group (*n* = 12). *Significant difference compare to the normal controls, †Significant difference compare to the PBS-injected group, *P* < 0.05. Scale bars: 2.5 mm.

After BMSC transplantation, skin lesion areas in E20 NTD fetuses (*n* = 23) were reduced by 29.9 ± 5.6%, which was significantly different from the control group (*n* = 30; *P* < *0.01*) (Fig. 7a–d). In some fetuses with high BMSC engraftment, complete repair of the skin lesion was observed (Fig. 7a). To determine whether BMSC transplantation improved neurological function in NTD fetuses, we monitored motor-evoked potentials (MEPs) in E20 fetuses by transcranial electrical stimulation (Fig. 7e). MEPs reflect the integrity of spinal cord motor pathways^42^ and may predict long-term recovery in animals and possibly humans^43^. We observed that the BMSC-treated group exhibited a significantly shorter latency and significantly higher MEP amplitude than the PBS-injected group, indicating that the transplanted fetuses presented a significantly improved motor neurological outcome (Fig. 7f–i).

## Discussion

Although prenatal diagnosis of NTDs allows for early intervention, NTD treatment options are still limited and unsatisfactory. Currently, both prenatal and postnatal NTD surgery focuses only on the repair of the unclosed neural tube rather than the regeneration of damaged neurons, which may be associated with unsatisfactory neurological recovery. Here, we used ex vivo and in vivo embryonic/fetal rat NTD models to demonstrate that transamniotic BMSC transplantation can repair lesions associated with an open neural tube and improve neurological function via lesion-specific engraftment and regeneration.

Our established ex vivo embryonic rat NTD model uses a WEC system and atRA exposure, allowing transamniotic BMSC injection and real-time tracking of transplanted BMSCs in rat embryos as early as E10.5. We demonstrated that BMSCs specifically migrated into and repaired defective neural tubes in early NTD embryos, which was confirmed using an in vivo model. Compared to previous studies showing that donor MSCs migrate mainly into the fetal bone marrow, placenta, and umbilical cord^30–32,35,36^, the trafficking patterns of the donor BMSCs after transamniotic injection reported here were different. These differences may have been caused by multiple factors, including the passage number and source of MSCs, culture conditions, cell number, time points of MSC delivery, labeling method of donor MSCs, and needle for cell injection. Our results further support the use of the amniotic cavity approach for transplanting stem cells for the treatment of congenital malformations in embryos/fetuses.

Directional migration, engraftment, and survival of BMSCs are essential for the efficacy of transamniotic BMSC-based therapies for NTDs. Given the potential clinical translational value of this treatment, we performed RNA-seq to explore the key genes that mediate spontaneous BMSC migration into the defective neural tube. Results showed that the most significantly upregulated migration-related genes in NTD embryos were related to cell motility and cell-cell adhesion. Moreover, the expression of *c-Met*, *Arhgap27*, *Nexn*, *Myl7*, and *Mylpf* in the focal adhesion pathway, *Prkch*, *Myh6*, and *Myh7* in the tight junction pathway, and *Col5a*, *Lamc2*, and *Itga8* in the ECM-receptor interaction pathway was significantly upregulated in NTD embryos. Therefore, the differentially expressed genes of these pathways may participate in BMSC directional migration.

HGF and its receptor c-MET are a ligand receptor pair. Based on their known functions, the HGF/c-MET signaling pathway is suspected to play a dual role, both recruiting BMSCs to damaged tissues^44,45^ and promoting nerve regeneration^17,46^. Abe et al.^37^ reported that transamniotic afMSC therapy promotes HGF secretion in the spina bifida lesion, indicating that HGF plays an important role in MSC transplantation for NTD therapy. Our study also showed that high HGF expression was mainly detected in the engrafted BMSCs observed in NTD regions that underwent BMSC repair. Furthermore, a concordant increase in the c-MET expression was observed at the dorsal surface of the defective neural tube. This phenomenon indicates that the high c-MET-expressing malformed neural tubes might recruit the transplanted BMSCs that present high HGF levels. Our intervention experiments with an HGF neutralizing antibody and c-MET inhibitor demonstrated that the interaction between HGF and c-MET was indeed associated with the migration of BMSCs into the defective spinal cord.

Compared to the findings of our previous study that used direct spinal column BMSC injection^20^, transamniotic BMSC administration showed a more widespread cell distribution in defective fetuses and a better effect on defective skin repair. In some NTD fetuses, a complete repair of the skin lesion was observed after BMSC transplantation, which protected the previously exposed neural tissue from stimulation by the amniotic fluid. Indeed, most BMSCs engrafted in the damaged skin expressed K19. These data suggest that transamniotically transplanted BMSCs not only covered the exposed neural tissue but also differentiated into epidermal stem cells to promote the regeneration of rudimentary skin in the defective region. Previous studies have reported that BMSCs promote dermal repair in chronic wounds, burns, and diabetic wounds^47–49^. In our previous studies, we also demonstrated that BMSCs injected directly into a damaged spinal column expressed early neuronal markers^20–22^. As the early transplantation time allowed transamniotic injection, we focused on the expression of some mature neuronal markers in this study. Our results collectively suggest that following transamniotic injection, BMSCs that engrafted in the neural tissue promoted the regeneration of sensory and motor neurons and formation of synapses, which is crucial for neural function recovery. Moreover, BMSCs originating from the mesodermal germ layer are classically described as cells generating different mesenchymal cell lineages^48,49^. However, many reports have suggested that BMSCs can transdifferentiate into non-mesenchymal lineages, including neurons, epithelial cells, and endothelial cells both in vitro and in vivo^47–56^. Compared to transamniotic NSC therapy, BMSCs with multi-lineage differentiation ability repaired more types of damaged tissues resulting from spina bifida. The mechanisms of BMSC transdifferentiation observed in these studies are unclear but appear to be regulated by multiple factors, including different microenvironments.

Transplanted BMSCs exhibit a plastic ability to respond specifically to different microenvironments^40,53^. For example, after engraftment in the normal fetal mouse brain, BMSCs can express nestin (neuroepithelial marker) in the subventricular zone, βIII-tubulin (early neuronal marker) in the midbrain, tau and MAP2 (mature neuronal markers) in the neocortical layers, and GFAP (astrocytic marker) in the pons and basal ganglia^53^. The high potential of differentiation of BMSCs into neurons and skin cells observed in our NTD model may be due to early embryos were in a rapid development stage and lacked robust immune systems, resulting in a beneficial microenvironment for the engraftment and transdifferentiation of transplanted cells^57^. Our previous study showed that the prenatal rat spinal cord microenvironment was more conducive to neural differentiation of transplanted BMSCs than the postnatal rat spinal column one^21^. In addition, the therapeutic efficacy of MSCs depends not only on their direct local differentiation but also on other mechanisms, such as their paracrine capacity.

Altogether, transamniotic delivery of BMSCs repaired multiple tissue defects and improved neural function recovery in the rat NTD model. This was achieved by site-specific migration and differentiation of BMSCs and other mechanisms triggered by donor cell delivery, which in combination promoted neural and epidermal tissue repair in the defective neural tube. Furthermore, transamniotically transplanted BMSCs covered and protected the exposed neural tissue in the defective area and further promoted neural function regeneration in NTD fetuses. Our findings provide new options to tackle the unsatisfactory neural repair associated with intra-uterine fetal surgery to treat NTDs. This BMSC delivery strategy is less traumatic for both mothers and fetuses and might provide a feasible approach for correcting malformations in fetuses. Also, it may be beneficial for treating multiple malformations simultaneously. Thus, transamniotic stem cell transplantation has broad clinical application prospects. In the future, clinical trials will be needed to evaluate the efficacy and safety of this stem cell transplantation approach.

## Material and methods

### Experiment animals

Outbred Wistar rats (10 to 12 weeks old, 250 to 300 g; 4 weeks old, about 100 g) were purchased from the Animal Center of China Medical University. All rats were supplied with food and water ad libitum and kept under pathogen-free conditions with a 12-h light/dark cycle. The rats were mated overnight. The morning that the vaginal plug was observed was considered gestational day 0 (E0). All procedures adhered to the National Institute of Health Guide for the Care and Use of Laboratory Animals and were approved by the Committee for Animal Care at China Medical University.

### Isolation, culture expansion, and transfection of BMSCs

BMSCs were isolated from the bone marrow of 4-week-old Wistar rats, expanded and identified the phenotypes by specific antibodies CD90, CD44, CD73, CD29, CD34, and CD45 as previously reported [20]. Briefly, BMSCs were cultured in DMEM/F12 (HyClone, USA) supplemented with 10% fetal bovine serum (FBS; Gibco, USA), 100 IU/mL penicillin and 100 μg/mL streptomycin (Gibco, USA). Primary isolated BMSCs were defined as P0. At confluency, cells were passaged and P3-4 were used for transplantation. To visualize the transplanted BMSCs, cells were transfected with eGFP-expressing adeno-5 vector (100 pfu/cell; SinoGenoMax.Co., Ltd, China) 24 h before transplantation. The transfected BMSCs were trypsinized, centrifuged, and resuspended in PBS.

### WEC, atRA treatment, and transamniotic BMSC injection

Pregnant females were euthanised at E10, and embryos were dissected from the uterus and cultured according to Buckley et al.^58^. Briefly, embryos with intact yolk sacs and ectoplacental cones were placed in sealed culture bottles (three embryos/bottle) containing 3 mL sterile heat-inactivated rat serum supplemented with 2 mg/mL glucose. Culture bottles were placed in a roller apparatus and rotated at 25 rpm in a 37 °C incubator with continuous supplement of a gas mixture, including different concentrations of O_2_ (5% O_2_ for the first 18 h, 20% O_2_ from 19 h to 36 h, and 60% O_2_ from 37 h to 48 h) and 5% CO_2_, balanced with N_2_.

To induce NTDs in the cultured embryos, 0.1% DMSO (v/v; control cultures) or 1, 3, or 10 μM atRA (Sigma-Aldrich, USA)) was added to the culture medium. After 12 h, embryos were transferred to fresh medium and cultured for another 36 h. After culturing, the amniotic cavity was incised, and embryos were examined for the presence of NTDs using a stereomicroscope (M165FC, Leica, Germany). NTDs were classified as isolated spina bifida, isolated exencephaly, or a combination of both defects.

For the ex vivo transamniotic injection (Fig. S3), GFP^+^ BMSCs or PBS were injected into the amniotic cavity through the dorsal aspect of the embryo, while avoiding the embryo, blood vessels of the yolk sac, and placenta, using a glass micropipette connected to a Hamilton syringe under a stereomicroscope. Approximately 500 cells (in 0.2 μL PBS) were injected per embryo. Micropipettes for injection with a tip diameter < 50 μm were made from borosilicate glass capillaries (1 mm diameter; model GD-1; Narishige Scientific Instruments, Japan) on a micropipette puller (model PB-7; Narishige Scientific Instruments, Japan). After culture, the engraftment and distribution of GFP^+^ BMSCs in the embryos were examined, and images were captured with a DS-Qi2 CCD camera (NY-1S35, Nikon, Japan) (Fig. S3).

### Time-lapse tracking of GFP^+^ BMSCs

Time-lapse tracking was used to monitor the movement of BMSCs. GFP^+^ BMSCs were seeded in 35 mm glass bottom dishes, allowed to attach overnight, and co-cultured with E10.5 embryos containing atRA-induced NTDs, which had the yolk sacs removed, in a humidified environment microscope/stage incubator (5% CO_2_ at 37 °C) mounted on a Delta Vision Elite High-Resolution Microscope (Applied Precision). The movement of 35 randomly selected cells was recorded at 7 min intervals for 24 h using the DeltaVision Elite High-Resolution Imaging System (GE Healthcare Life Sciences, USA). Migration distances of individual cells were determined and analyzed. The shortest distance between BMSCs and the embryo was measured at 0, 6, 12, and 16 h. The location of one cell at 0 h points to that at 16 h, which is defined as the motion vector of the cell. According to the motion vectors, cells were divided into three modes: quiescent, deviated from the embryo, and close to the embryo. If a cell′s motion vector had component on the line of the shortest distance between the cell and the embryo at 0 h, it was considered to belong to the mode of movement close to the embryo.

### RNA-seq analysis

Total mRNA was isolated from the spinal neural tubes of normal and NTD embryos using an RNeasy mini kit (Qiagen, Hamburg, Germany). Three neural tubes were used for each group, and equal amounts of RNA from three individual neural tubes were pooled. RNA sequencing libraries were constructed using the Illumina mRNA-seq Prep Kit Gene expression levels were calculated using the RPKM (Reads Per kb per Million reads) method. We used “false discovery rate ≤0.001 (FDR ≤ 0.001) and the absolute value of log2Ratio ≥ 1” as the threshold to judge the significance of gene expression difference. The KEGG database (http://www.genome.jp/kegg/) was used to classify and group the identified genes. KEGG pathway analysis was performed using the NCBI FLink site.

### RT-qPCR analysis

RT-qPCR was performed according to standard procedures. Briefly, total RNA was isolated, and cDNA synthesis involved use of 2 μg RNA with the TaKaRa RNA PCR kit (Takara, Tokyo, Japan). Real-time PCR amplifications were performed in triplicate using the Lightcycler® 480 Instrument (Roche, Mannheim, Germany) with primers in Table S3. The relative mRNA levels of each sample were calculated according to the 2^−ΔΔCt^ method using GAPDH expression for normalization.

### Anti-HGF antibody and c-MET inhibitor treatment of transamniotic injected BMSCs ex vivo

To investigate if HGF/c-MET signaling affected BMSC engraftment into NTD embryos, cultured BMSCs were incubated with the function blocking anti-HGF antibody (5 ng/2 × 10^4^ cells; Abcam, USA) for 24 h prior to use. The c-MET inhibitor PHA-665752 (128 mg/mL dissolved in DMSO, 25 mg/kg body weight; Selleck, USA) was injected into amniotic cavity of embryos 24 h prior to BMSC transplantation ex vivo. The embryos with or without c-MET inhibitor treatment were cultured for 48 h after BMSC or HGF antibody-treated BMSC transplantation. The embryos were frozen-sectioned into serial sections and used for cell counting.

### In utero transamniotic BMSC injection

For the in utero study, spina bifida aperta was induced with a single intra-gastric gavage of atRA (4% wt/vol in olive oil; 140 mg/kg body weight) to pregnant rats on E10 as previously described^20,59^.

In utero transamniotic BMSC microinjection was performed on E15 embryos. Pregnant rats were anesthetized with pentobarbitone sodium (40 mg/kg body weight). An incision was made in the abdominal wall, and the uterus was exteriorized. Under the operation microscope (Moller, Germany), the embryos with a uniform position and size of spina bifida were chosen and randomly divided into BMSC-injected and PBS-injected groups. Using a glass micropipette connected to a Hamilton syringe, 2 μL of GFP^+^ BMSC suspension (5 × 10^6^ cells) or PBS was injected into the amniotic fluid via the uterine wall by the ventral aspect of the embryos, with care to avoid the embryos, placenta, and umbilical cord. After injections, the uterus was returned to the abdomen, and the abdominal wall was closed. The pregnant rats recovered from the anesthesia within 1 h and were returned to their home cage. The pregnant rats were euthanized at E20 by an overdose of pentobarbitone sodium, and the injected fetuses were harvested for analysis.

### In vivo imaging and fluorescence imaging

The fetuses were anesthetized by immersion in cold physiological saline at 10 °C on E20, and the skeletal development and distribution of GFP^+^ cells in whole fetuses were detected using an in vivo image system (MS FX PRO, Carestream Health, KODAK, USA). The dorsal skin images of the fetuses were taken using a fluorescence stereomicroscope (M165FC, Leica, Germany) fitted with a Nikon DS-Qi2 digital camera (NY-1S35, Nikon, Japan). Measurements of the skin lesion area of the fetuses were made using BR analysis software.

### Tissue preparation, BMSC counting, and immunofluorescence

The embryos treated ex vivo or different organs isolated from E20 fetuses treated in vivo were fixed in freshly prepared 4% paraformaldehyde, and were then cryoprotected in 20% sucrose for 24 h, embedded in Optimal Cutting Temperature compound, and sectioned into 30-µm serial sections using a freezing microtome (Microm hm525, Thermo, Germany). GFP^+^ cells were located and counted by fluorescence microscopy (80i, Nikon, Japan) of serial sections of whole fetuses or various tissues. All sections were counterstained with DAPI. Only those GFP^+^ cells that contained a nucleus were counted to avoid multiple counting of the same cell. The BMSC engraftment rates were calculated as engrafted cell numbers/total injected cell numbers multiplied by 100. Sections with GFP^+^ cells were marked and kept at −80 °C in the dark for further immunofluorescence analysis.

The primary antibodies used for immunofluorescence were rabbit anti-GFP (1:200; AG279; Beyotime Institute of Biotechnology, China), TBX1 (1:100; QC2875, Sigma, USA), ITGA1 (1:200; ab134179, Abcam, USA), c-MET (1:200; #8198, Cell Signaling, USA), HGF (1:200; ab83760, Abcam, USA), ISLET1 (1:200; ab20670, Abcam, USA), SYN (1:200; AB1543P, Millipore, Germany), or mouse anti-GFP (1:200; AG281; Beyotime Institute of Biotechnology, China), BRN3A (1:50; MAB1585, Millipore, Germany), K19 (1:100; sc-376126; Santa, China), and SYT (1:200; MAB5200, Millipore, Germany). The secondary antibodies included Alexa Fluor 488-conjugated goat anti-rabbit or anti-mouse IgG antibody (1:100; Invitrogen, USA) and TRITC-conjugated goat anti-mouse or anti-rabbit IgG antibody (1:50; Millipore, Germany). The immunofluorescence analysis was performed according to standard procedures. Images were taken with a C1 confocal microscope (Nikon, Japan). To determine the percentage of transplanted BMSCs that expressed specific cell markers, all GFP-positive cells that were also immunopositive for the indicated cell markers were counted in each section.

For cultured cell staining, BMSCs were seeded in 35-mm glass bottom dishes (In Vitro Scientific) and fixed with 4% paraformaldehyde for 30 min. The numbers of BMSCs expressing HGF, c-MET, or K19 were counted by blinded observers. Ten random fields (40× magnification) were analyzed. The percentages of BMSCs expressing specific markers were reported as the number of double-positive cells/total number of GFP^+^ cells/field.

### Electrophysiological tests

Fetuses (E20) were anaesthetized with pentobarbitone sodium (40 mg/kg body weight) and placed prone on a temperature-controlled plate to maintain skin temperature above 32 °C. A multi-channel electrophysiology recorder (PL3508/P, Powerlab, Australia) was used for testing. MEPs were elicited by transcranial stimulation with two needle electrodes: the anode was placed over the sensorimotor cortex, whereas the cathode was placed on the nose. Single electrical pulses of 3 mA intensity at 1 Hz and 100 μs duration were applied, and MEPs were recorded with monopolar needle electrodes from the anterior tibialis muscles. The active recording needle electrode was inserted in the tibialis anterior muscles with a reference electrode placed at the fourth toe and a ground electrode at the base of the tail. To ensure reproducibility of the electrophysiological tests, stimulation and recording needles were placed at the same locations in all fetuses. Signals were amplified, filtered (band-pass 1–5000 Hz), and displayed on the monitor. The latency to onset and amplitude from peak-to-peak of the largest positive-negative deflection was measured. Fifteen consecutive responses were recorded with a time interval of 3 s between stimuli. The recording with the highest amplitude was used for analysis.

### Statistical analysis

All analyses were performed in a double-blind manner. All data are expressed as the mean ± SEM. The Student′s *t*-test was used for single comparisons. One-way ANOVA followed by the Bonferroni′s post-test was used for comparisons of more than two groups. The Pearson correlation was calculated with SPSS 16.0. *P*-values < 0.05 were considered to be statistically significant.

## Supplementary information

Supplementary figure legends

Figure S1

Figure S2

Figure S3

Table S1

Table S2

Table S3

Video S1

Video S2
