# Supplementary material for: Transamniotic mesenchymal stem cell therapy for neural tube defects preserves neural function through lesion-specific engraftment and regeneration
Source: Cell Death Dis. 2020 Jul 13;11(7):523. doi: 10.1038/s41419-020-2734-3 (PMC7354991; doi:10.1038/s41419-020-2734-3)
Supplement: Supplementary file 1 — Supplementary figure legends [file 41419_2020_2734_MOESM1_ESM.docx]

**Fig. S1 Morphology and distribution of BMSCs. A-C.** The transplanted BMSCs mainly migrated to the defective regions of the neural tubes, and of these, the most common areas of BMSC engraftment were the roof plate of the defective neural tube (A), peripheral mesenchyma (B) and the ganglions (C). **D.** Transplanted BMSCs (GFP labeled) presented with various shapes, such as triangular, round, stellate, long and spindle, *in vivo*. **E.** The GFP-labeled BMSCs cultured *in vitro* were mostly of spindle shape. Scale Bars: 100μm.

**Fig. S2 Screening of donor cells in the fetuses after intra-amniotic BMSC transplantation.** **A.** Representative sections of different tissues from the fetuses received intra-amniotic BMSC transplantation. Scale bars: 500 μm. GFP^+^ BMSCs were only observed on the defective spinal cord and skin. **B.** Quantitative analysis of *Gfp* expression in different sites of intra-amniotic transplanted fetuses on E20 by RT-qPCR (n=5).

**Fig. S3 Whole embryo culture (WEC) and intra-amniotic BMSC injection. A.** The uterus was dissected from the pregnant rat. **B.** The wall of the uterus was opened. **C.** The fetal sac containing the fetal rat was exposed. **D.** The decidua was removed from the uterus, and Reichert’s membrane was removed carefully. **E.** The embryo with intact yolk sac and ectoplacental cones was freed. **F.** BMSCs expressing GFP were injected into the amniotic cavity. **G.** The fetal sac observed under a fluorescence microscope after BMSC injection. **H.** The fetal sac and embryo cultured for 48 hrs. **I.** The normal rat embryo derived from the amniotic sac in picture
