# Supplementary figures and images for: Transamniotic mesenchymal stem cell therapy for neural tube defects preserves neural function through lesion-specific engraftment and regeneration
Source: Cell Death Dis. 2020 Jul 13;11(7):523. doi: 10.1038/s41419-020-2734-3 (PMC7354991; doi:10.1038/s41419-020-2734-3)

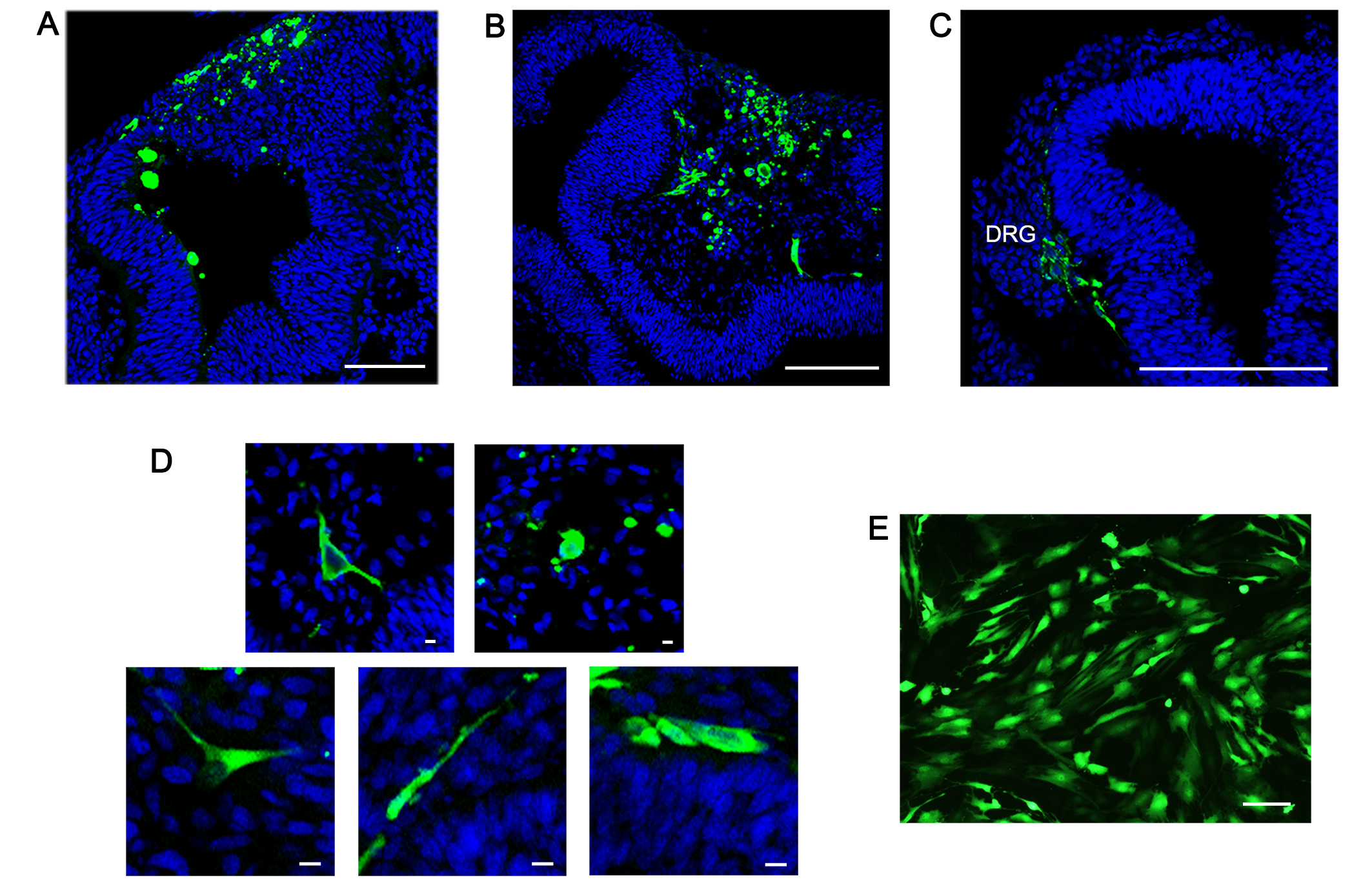

Supplement: Supplementary file 2 — Figure S1 [file 41419_2020_2734_MOESM2_ESM.tif]

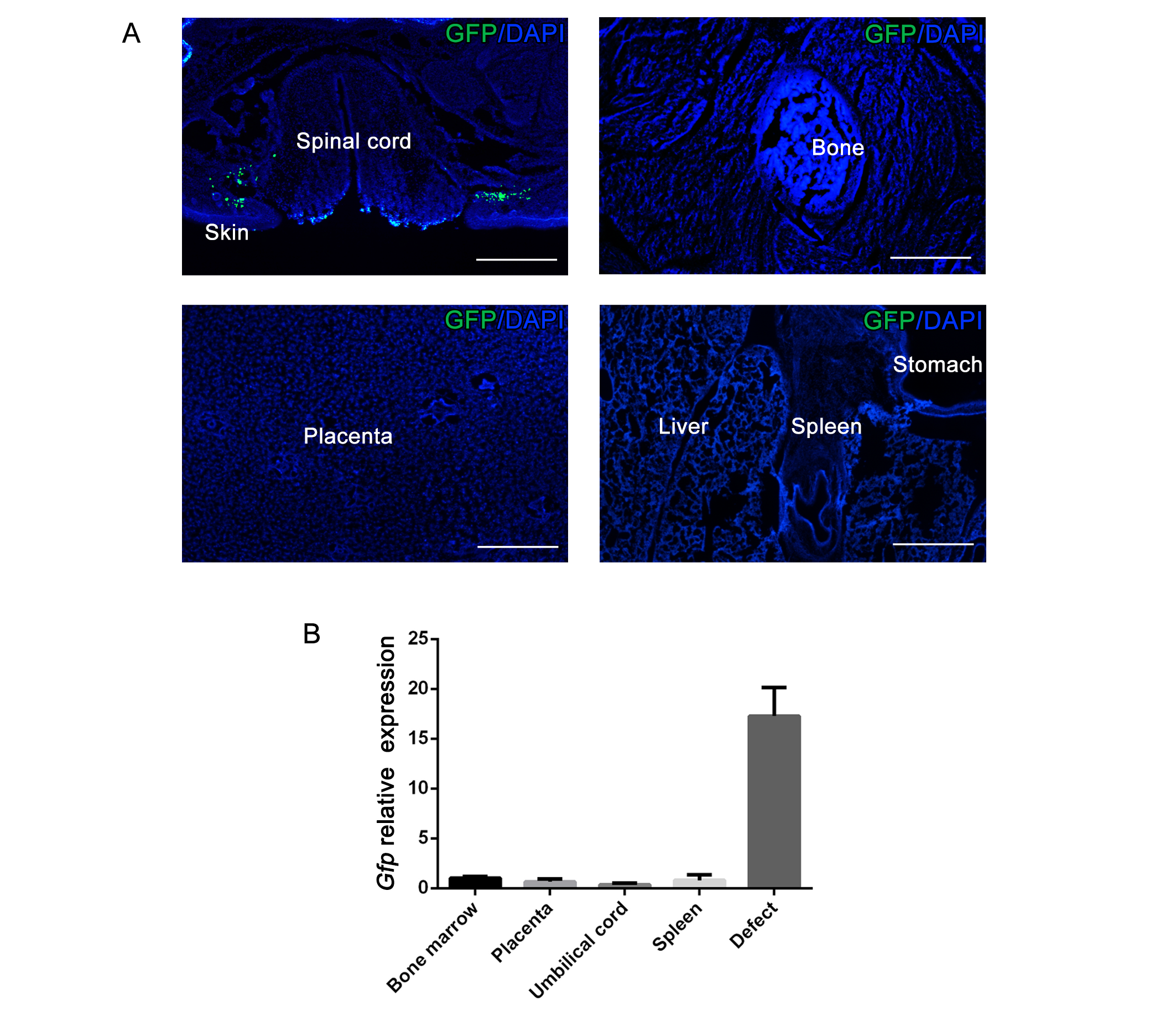

Supplement: Supplementary file 3 — Figure S2 [file 41419_2020_2734_MOESM3_ESM.tif]

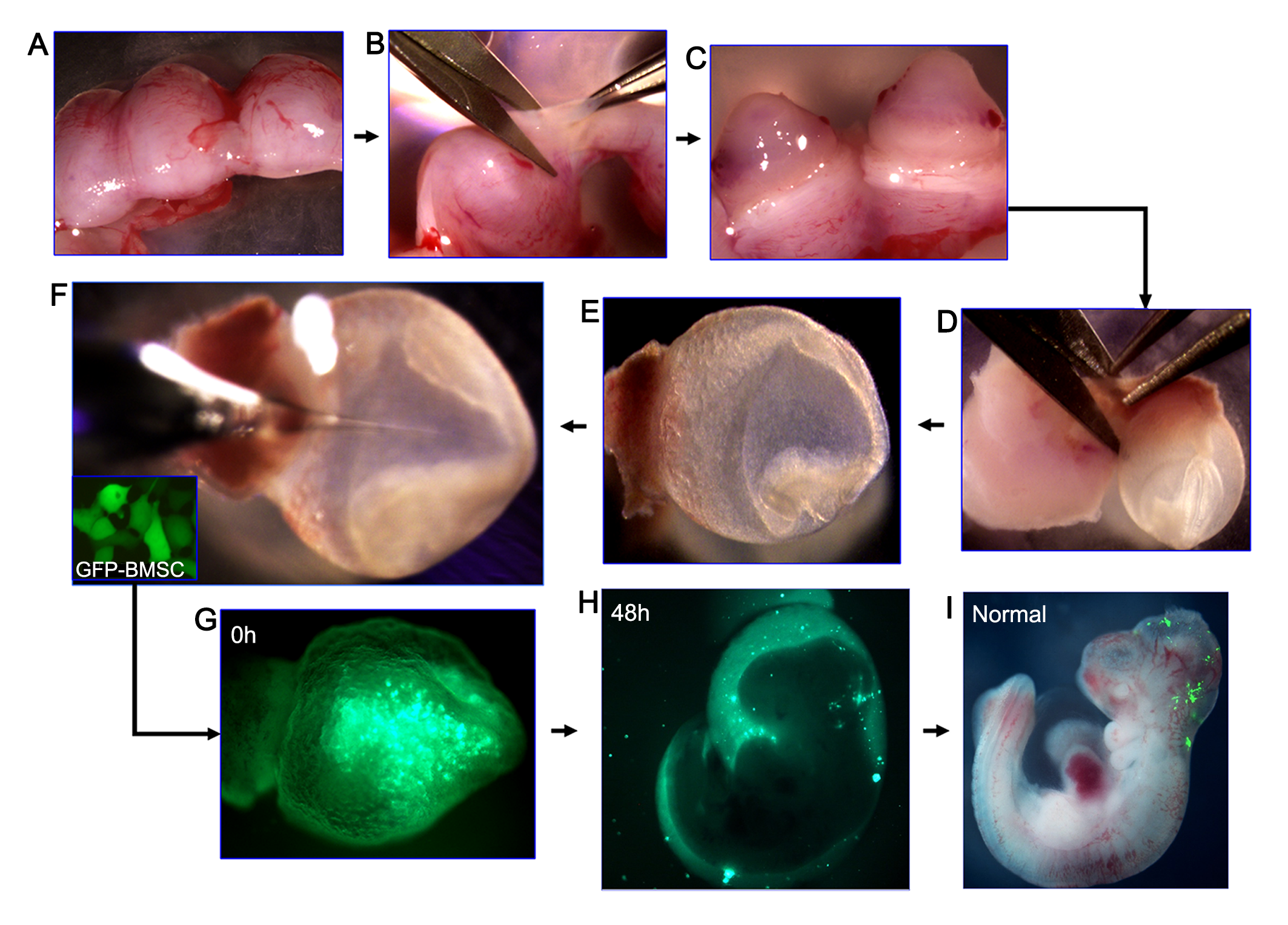

Supplement: Supplementary file 4 — Figure S3 [file 41419_2020_2734_MOESM4_ESM.tif]
