# Supplementary material for: Transamniotic mesenchymal stem cell therapy for neural tube defects preserves neural function through lesion-specific engraftment and regeneration
Source: Cell Death Dis. 2020 Jul 13;11(7):523. doi: 10.1038/s41419-020-2734-3 (PMC7354991; doi:10.1038/s41419-020-2734-3)
Supplement: Supplementary file 5 — Table S1 [file 41419_2020_2734_MOESM5_ESM.docx]

| **Table. S1 The influence of intra-amniotic injection for the embryo dysmorphology.** | | | | | |
| --- | --- | --- | --- | --- | --- |
|  | **No injected group** | **PBS-injected group** | **BMSC-injected group** | |  |
| Crown-rump length (1mm) | 5.30±0.12（12） | 5.11±0.16（12） | 5.09±0.12（12） |  | |
| Head length (mm) | 3.06±0.14（12） | 3.15±0.15（12） | 3.17±0.16（12） |  | |
| Yolk sac diameter (mm) | 5.84±0.10（12） | 5.71±0.13（12） | 5.90±0.39（12） |  | |
| Somite number | 30.50±2.62（12） | 30.75±1.86（12） | 30.25±1.82（12） |  | |
| Total morphological score | 56.85±1.76（12） | 55.58±2.61（12） | 56.08±1.88（12） |  | |

Values are expressed as mean ± SEM for Crown-rump length, Head length, Yolk sac diameter, Somite number and Total morphological score.
