# Supplementary material for: Transamniotic mesenchymal stem cell therapy for neural tube defects preserves neural function through lesion-specific engraftment and regeneration
Source: Cell Death Dis. 2020 Jul 13;11(7):523. doi: 10.1038/s41419-020-2734-3 (PMC7354991; doi:10.1038/s41419-020-2734-3)
Supplement: Supplementary file 6 — Table S2 [file 41419_2020_2734_MOESM6_ESM.docx]

| **Table. S2 Disregulated KEGG pathways in the exancephaly embryos.** | | | | | | | |
| --- | --- | --- | --- | --- | --- | --- | --- |
| **No** | **Pathway** | **Up-regulated genes number** | **Down-regulated genes number** | **DEGs with pathway annotation (285)** | **All genes with pathway annotation (18766)** | **P value** | **Pathway ID** |
| 1 | [Focal adhesion](file:///E:\experiment\result\20141209\20131113RNA-seq\RNA-seq差异基因\KEGG.xlsx#RANGE!gene2) | 15 | 3 | 18 (6.32%) | 459 (2.45%) | 0.0002 | ko04510 |
| 2 | [Dilated cardiomyopathy](file:///E:\experiment\result\20141209\20131113RNA-seq\RNA-seq差异基因\KEGG.xlsx#RANGE!gene9) | 14 | 1 | 15 (5.26%) | 445 (2.37%) | 0.0035 | ko05414 |
| 3 | [Regulation of actin cytoskeleton](file:///E:\experiment\result\20141209\20131113RNA-seq\RNA-seq差异基因\KEGG.xlsx#RANGE!gene14) | 14 | 2 | 16 (5.61%) | 578 (3.08%) | 0.0157 | ko04810 |
| 4 | [Hypertrophic cardiomyopathy (HCM)](file:///E:\experiment\result\20141209\20131113RNA-seq\RNA-seq差异基因\KEGG.xlsx#RANGE!gene1) | 13 | 2 | 15 (5.26%) | 329 (1.75%) | 0.0002 | ko05410 |
| 5 | [Transcriptional misregulation in cancer](file:///E:\experiment\result\20141209\20131113RNA-seq\RNA-seq差异基因\KEGG.xlsx#RANGE!gene4) | 13 | 5 | 18 (6.32%) | 484 (2.58%) | 0.0005 | ko05202 |
| 6 | [Tight junction](file:///E:\experiment\result\20141209\20131113RNA-seq\RNA-seq差异基因\KEGG.xlsx#RANGE!gene13) | 12 | 2 | 14 (4.91%) | 481 (2.56%) | 0.0157 | ko04530 |
| 7 | [Vascular smooth muscle contraction](file:///E:\experiment\result\20141209\20131113RNA-seq\RNA-seq差异基因\KEGG.xlsx#RANGE!gene6) | 11 | 3 | 14 (4.91%) | 386 (2.06%) | 0.0024 | ko04270 |
| 8 | [Pathways in cancer](file:///E:\experiment\result\20141209\20131113RNA-seq\RNA-seq差异基因\KEGG.xlsx#RANGE!gene16) | 11 | 6 | 17 (5.96%) | 642 (3.42%) | 0.0192 | ko05200 |
| 9 | [Axon guidance](file:///E:\experiment\result\20141209\20131113RNA-seq\RNA-seq差异基因\KEGG.xlsx#RANGE!gene8) | 9 | 3 | 12 (4.21%) | 316 (1.68%) | 0.0034 | ko04360 |
| 10 | [Cardiac muscle contraction](file:///E:\experiment\result\20141209\20131113RNA-seq\RNA-seq差异基因\KEGG.xlsx#RANGE!gene15) | 9 | 0 | 9 (3.16%) | 262 (1.4%) | 0.0191 | ko04260 |
| 11 | [Serotonergic synapse](file:///E:\experiment\result\20141209\20131113RNA-seq\RNA-seq差异基因\KEGG.xlsx#RANGE!gene5) | 7 | 3 | 10 (3.51%) | 203 (1.08%) | 0.0011 | ko04726 |
| 12 | [Malaria](file:///E:\experiment\result\20141209\20131113RNA-seq\RNA-seq差异基因\KEGG.xlsx#RANGE!gene3) | 6 | 2 | 8 (2.81%) | 116 (0.62%) | 0.0004 | ko05144 |
| 13 | [ECM-receptor interaction](file:///E:\experiment\result\20141209\20131113RNA-seq\RNA-seq差异基因\KEGG.xlsx#RANGE!gene21) | 6 | 1 | 7 (2.46%) | 203 (1.08%) | 0.0358 | ko04512 |
| 14 | [Dopaminergic synapse](file:///E:\experiment\result\20141209\20131113RNA-seq\RNA-seq差异基因\KEGG.xlsx#RANGE!gene22) | 5 | 2 | 7 (2.46%) | 204 (1.09%) | 0.0366 | ko04728 |
| 15 | [Arrhythmogenic right ventricular cardiomyopathy (ARVC)](file:///E:\experiment\result\20141209\20131113RNA-seq\RNA-seq差异基因\KEGG.xlsx#RANGE!gene25) | 5 | 0 | 6 (2.11%) | 166 (0.88%) | 0.0414 | ko05412 |
| 16 | [MAPK signaling pathway](file:///E:\experiment\result\20141209\20131113RNA-seq\RNA-seq差异基因\KEGG.xlsx#RANGE!gene27) | 5 | 7 | 12 (4.21%) | 453 (2.41%) | 0.0447 | ko04010 |
| 17 | [GnRH signaling pathway](file:///E:\experiment\result\20141209\20131113RNA-seq\RNA-seq差异基因\KEGG.xlsx#RANGE!gene12) | 4 | 4 | 8 (2.81%) | 179 (0.95%) | 0.0061 | ko04912 |
| 18 | [Retrograde endocannabinoid signaling](file:///E:\experiment\result\20141209\20131113RNA-seq\RNA-seq差异基因\KEGG.xlsx#RANGE!gene10) | 3 | 4 | 7 (2.46%) | 132 (0.7%) | 0.0041 | ko04723 |
| 19 | [Phenylalanine metabolism](file:///E:\experiment\result\20141209\20131113RNA-seq\RNA-seq差异基因\KEGG.xlsx#RANGE!gene11) | 3 | 1 | 4 (1.4%) | 47 (0.25%) | 0.0056 | ko00360 |
| 20 | [Cell adhesion molecules (CAMs)](file:///E:\experiment\result\20141209\20131113RNA-seq\RNA-seq差异基因\KEGG.xlsx#RANGE!gene17) | 3 | 5 | 9 (3.16%) | 273 (1.45%) | 0.0241 | ko04514 |
| 21 | [Glutamatergic synapse](file:///E:\experiment\result\20141209\20131113RNA-seq\RNA-seq差异基因\KEGG.xlsx#RANGE!gene18) | 3 | 4 | 7 (2.46%) | 187 (1%) | 0.0244 | ko04724 |
| 22 | [Tyrosine metabolism](file:///E:\experiment\result\20141209\20131113RNA-seq\RNA-seq差异基因\KEGG.xlsx#RANGE!gene20) | 3 | 1 | 4 (1.4%) | 80 (0.43%) | 0.0335 | ko00350 |
| 23 | [Cholinergic synapse](file:///E:\experiment\result\20141209\20131113RNA-seq\RNA-seq差异基因\KEGG.xlsx#RANGE!gene24) | 3 | 3 | 6 (2.11%) | 165 (0.88%) | 0.0404 | ko04725 |
| 24 | [Toxoplasmosis](file:///E:\experiment\result\20141209\20131113RNA-seq\RNA-seq差异基因\KEGG.xlsx#RANGE!gene26) | 3 | 4 | 7 (2.46%) | 212 (1.13%) | 0.0435 | ko05145 |
| 25 | [Melanogenesis](file:///E:\experiment\result\20141209\20131113RNA-seq\RNA-seq差异基因\KEGG.xlsx#RANGE!gene19) | 2 | 4 | 6 (2.11%) | 157 (0.84%) | 0.0329 | ko04916 |
| 26 | [Complement and coagulation cascades](file:///E:\experiment\result\20141209\20131113RNA-seq\RNA-seq差异基因\KEGG.xlsx#RANGE!gene28) | 2 | 4 | 6 (2.11%) | 172 (0.92%) | 0.0477 | ko04610 |
| 27 | [Cyanoamino acid metabolism](file:///E:\experiment\result\20141209\20131113RNA-seq\RNA-seq差异基因\KEGG.xlsx#RANGE!gene7) | 1 | 1 | 2 (0.7%) | 6 (0.03%) | 0.0033 | ko00460 |
| 28 | [Adipocytokine signaling pathway](file:///E:\experiment\result\20141209\20131113RNA-seq\RNA-seq差异基因\KEGG.xlsx#RANGE!gene23) | 1 | 4 | 5 (1.75%) | 121 (0.64%) | 0.0374 | ko04920 |
