# Supplementary material for: Transamniotic mesenchymal stem cell therapy for neural tube defects preserves neural function through lesion-specific engraftment and regeneration
Source: Cell Death Dis. 2020 Jul 13;11(7):523. doi: 10.1038/s41419-020-2734-3 (PMC7354991; doi:10.1038/s41419-020-2734-3)
Supplement: Supplementary file 7 — Table S3 [file 41419_2020_2734_MOESM7_ESM.docx]

| **Table S3. Primers used in the study.** | | | | |
| --- | --- | --- | --- | --- |
| **Genes** | **Accession number** | **Primer sequences (5’-3’) /exon location (nt)** | **Annealing Tm (°C)** | **PCR product(bp)** |
| *Prckh* | [NM_031085.2](https://www.ncbi.nlm.nih.gov/entrez/viewer.fcgi?db=nucleotide&id=52138606) | Sense: GCTCGCCAGAATAAAGGAGACAG/1297-1319 | 60 | 187 |
|  |  | Antisense:GACAAAGAACAGACGGTCAGGAGT/1460-1483 |  |  |
| *Birc3* | [NM_023987.3](https://www.ncbi.nlm.nih.gov/entrez/viewer.fcgi?db=nucleotide&id=347921973) | Sense: CAAGTTCAAGCTGGTTACCCTCATC/1229-1253 | 60 | 141 |
|  |  | Antisense:AGGTGTGTTCATCATCACTGCATC/1346-1369 |  |  |
| *Col5a3* | [NM_021760.1](https://www.ncbi.nlm.nih.gov/entrez/viewer.fcgi?db=nucleotide&id=11120709) | Sense: GGACTCACGGTGATGGGAAC/605-624 | 60 | 132 |
|  |  | Antisense:GGTCTCACAGTCAGGCAGGTAG/715-736 |  |  |
| *Prtg* | [NM_001037651.1](https://www.ncbi.nlm.nih.gov/entrez/viewer.fcgi?db=nucleotide&id=83320091) | Sense: TTGGGAACCAGAGCAGGAG/4318-4300 | 60 | 83 |
|  |  | Antisense:TGGAGCAACGGGAAAGGT/4365-4328 |  |  |
| *Myh8* | [NM_001100485.1](https://www.ncbi.nlm.nih.gov/entrez/viewer.fcgi?db=nucleotide&id=281604095) | Sense: GTTGGCTGGACAAGAACAAGG/1856-1876 | 60 | 103 |
|  |  | Antisense:CTGGCGTATGTGGAAAAGAGG/1938-1958 |  |  |
| *Itga 8* | [NM_001173972.1](https://www.ncbi.nlm.nih.gov/entrez/viewer.fcgi?db=nucleotide&id=291190714) | Sense: CCTTGGGAACCCGATGGT/2326-2343 | 60 | 71 |
|  |  | Antisense:TCTCAAGACGAGGAACAGCAAA/2375-2396 |  |  |
| *Lamc2* | [NM_001100640.1](https://www.ncbi.nlm.nih.gov/entrez/viewer.fcgi?db=nucleotide&id=281371493) | Sense:GCTCAGGGTGGTAATGGT/2216-2233 | 60 | 176 |
|  |  | Antisense:AGGCGGTTCTTGTAGTTGT/2373-2391 |  |  |
| *Hgf* | [NM_017017.2](https://www.ncbi.nlm.nih.gov/entrez/viewer.fcgi?db=nucleotide&id=291463303) | Sense:CACTCTTGACCCTGACACCC/961-980 | 60 | 172 |
|  |  | Antisense:CCAACGCTGACACGGAAT /1115-1132 |  |  |
| *c-Met* | [NM_031517.2](https://www.ncbi.nlm.nih.gov/entrez/viewer.fcgi?db=nucleotide&id=451958160) | Sense:ACCCAACCACGAGCACTG /1176-1193 | 60 | 211 |
|  |  | Antisense:GCGACCTTCTGATGTCCCTA/1367-1386 |  |  |
| *Angpt2* | [NM_134454.1](https://www.ncbi.nlm.nih.gov/entrez/viewer.fcgi?db=nucleotide&id=198278528) | Sense:TGGACCAGACCAGTGAAAT/595-613 | 60 | 139 |
|  |  | Antisense:GACACCAGCACCTGAAGC/716-733 |  |  |
| *Cdh8* | [NM_053393.2](https://www.ncbi.nlm.nih.gov/entrez/viewer.fcgi?db=nucleotide&id=186287287) | Sense:TCTTCACCGACTTACCTCC/1707-1725 | 60 | 173 |
|  |  | Antisense:GTTATCTTCCCATCATCTGC/1860-1879 |  |  |
| *Itga1* | [NM_001033998.2](https://www.ncbi.nlm.nih.gov/entrez/viewer.fcgi?db=nucleotide&id=146219850) | Sense:ATACCTTCCGTGCCATCA/763-780 | 60 | 121 |
|  |  | Antisense:TTGCCATTGTCACTTGCTT/865-883 |  |  |
| *Wnt7b* | [NM_001009695.1](https://www.ncbi.nlm.nih.gov/entrez/viewer.fcgi?db=nucleotide&id=57527451) | Sense:AGCCAACATCATCTGCAACA/99-118 | 62 | 122 |
|  |  | Antisense:GGCATTCATCGATACCCATC/201-220 |  |  |
| *Myh7* | [NM_017240.2](https://www.ncbi.nlm.nih.gov/entrez/viewer.fcgi?db=nucleotide&id=451958092) | Sense:TCCACCCCACATCTTCTC/458-475 | 60 | 154 |
|  |  | Antisense:AATGGCAGCAATAACAGC/594-611 |  |  |
| *Actn2* | [NM_001170325.1](https://www.ncbi.nlm.nih.gov/entrez/viewer.fcgi?db=nucleotide&id=281332156) | Sense:TTCATCGTCCACAGCATT/1816-1833 | 61 | 129 |
|  |  | Antisense:CACCTTCTCCACCTCATTC/1926-1944 |  |  |
| *Pcdh12* | [NM_053944.1](https://www.ncbi.nlm.nih.gov/entrez/viewer.fcgi?db=nucleotide&id=281371357) | Sense:AACCACCGAGGAAATA/3283-3268 | 60 | 265 |
|  |  | Antisense:TAGTGAGGGGCAATGACA/3515-3532 |  |  |
| *Fat2* | [NM_022954.1](https://www.ncbi.nlm.nih.gov/entrez/viewer.fcgi?db=nucleotide&id=12621131) | Sense:GGTGACAGTGGGGACATT/4644-4661 | 62 | 174 |
|  |  | Antisense:ACATTTGCCTCATAACGAA/4799-4817 |  |  |
| *Hhex* | [NM_024385.1](https://www.ncbi.nlm.nih.gov/entrez/viewer.fcgi?db=nucleotide&id=13242290) | Sense:CGACTACACGCACGCCCTAC/338-357 | 60 | 104 |
|  |  | Antisense:TCACTTGACCGCCTTTCCTTT/421-441 |  |  |
| *Tbx1* | [NM_001108322.1](https://www.ncbi.nlm.nih.gov/entrez/viewer.fcgi?db=nucleotide&id=157823230) | Sense:CCGCTTGTAAGTGCCTTTGCTCG/1036-1058 | 60 | 78 |
|  |  | Antisense:CACATCTTTGTCCGCTCCGTTGG/1091-1113 |  |  |
| *Arhgap27* | [NM_198759.1](https://www.ncbi.nlm.nih.gov/entrez/viewer.fcgi?db=nucleotide&id=38454257) | Sense:TACGAGGCTATCCCGGACTTG/201-621 | 60 | 94 |
|  |  | Antisense:TCTCTACGTTGGCATACACTGGTTC/670-694 |  |  |
| *Mylpf* | [NM_012605.2](https://www.ncbi.nlm.nih.gov/entrez/viewer.fcgi?db=nucleotide&id=158341640) | Sense:GAGCTACTGCCTTGCCCTCA/9-28 | 60 | 172 |
|  |  | Antisense:TGTCAATAATGCCATCCCTGTTC/158-180 |  |  |
| *Lrrtm1* | [NM_001109374.1](https://www.ncbi.nlm.nih.gov/entrez/viewer.fcgi?db=nucleotide&id=157820118) | Sense:TCCATATTCAGGAATCTGAGAGTGT/2453-2477 | 60 | 140 |
|  |  | Antisense:TGAAATTTCTGCTCCCACCTT/2572-2592 |  |  |
| *Ldb3* | [NM_001277166.1](https://www.ncbi.nlm.nih.gov/entrez/viewer.fcgi?db=nucleotide&id=461496475) | Sense:TCCAGCCAACGCCGACTA/415-423 | 60 | 95 |
|  |  | Antisense:CCAAGCCCTTCACCTCAAT/491-509 |  |  |
| *Ptprc* | [NM_001109890.2](https://www.ncbi.nlm.nih.gov/entrez/viewer.fcgi?db=nucleotide&id=1050109415) | Sense:AACTCGTTGAAAGGGATGA/1493-1511 | 60 | 142 |
|  |  | Antisense:AATACCCGTGGAATGCTC/1617-1634 |  |  |
| *Cntnap3* | NM_012601.2 | Sense:TTTTAGCCAACCAGAGCAT/963-981 | 60 | 84 |
|  |  | Antisense:CACAGCCCATCGTTCAGTA/898-916 |  |  |
| *Myl7* | NM_001106017.1 | Sense:GGAGGGTAAGTGTCCCAGAGG/198-218 | 60 | 154 |
|  |  | Antisense:GGGTCAAACATTCGGAAAGCA/331-351 |  |  |
| *Angpt1* | NM_053546.1 | Sense:AACCGAGCCTACTCACAG/1435-1452 | 60 | 153 |
|  |  | Antisense:GTCGTTATCAGCGTCCTT/1570-1587 |  |  |
| *Ttyh1* | NM_001106225.1 | Sense:CTCATCTTCATCGCTGTCT/268-286 | 60 | 176 |
|  |  | Antisense:TCACTGGTCTCGCTGTTAC/425-443 |  |  |
| *Nckap11* | NM_001108119.1 | Sense:CAAAGGCTGTGAGTGGAG/631-648 | 61 | 105 |
|  |  | Antisense:TGCTGATGAGGCTTAGGA/718-735 |  |  |
| *Hoxd4* | NM_001105885.1 | Sense:ATTGTCACCGAGCCTACC/954-971 | 60 | 171 |
|  |  | Antisense:GATAATCTTCGCTTTTGTCTT/1104-1124 |  |  |
| *Icam2* | NM_001007725.1 | Sense:TTACTTTGCCATTTCACTTG/665-684 | 61 | 121 |
|  |  | Antisense:CTTCACCCATAAACACTCG/767-785 |  |  |
| *Hoxd3* | NM_001271038.1 | Sense:TGAAGGAATCCCGACAGA/872-889 | 60 | 170 |
|  |  | Antisense:GCACAGGTAGCGGTTGAA/1024-1041 |  |  |
| *Gapdh* | NM_017008.4 | Sense: TGCCGCCTGGAGAAACCTGC/808-827 | 60 | 168 |
|  |  | Antisense: AGCAATGCCAGCCCCAGCAT/956-975 |  |  |
| *Gfp* | XM_013480425.1 | Sense: CTTCAAGATCCGCCACAACATC/576-597 | 62 | 165 |
|  |  | Antisense: ACCATGTGATCGCGCTTCTC/721-740 |  |  |
